# Clinical characteristics and prognostic analysis of patients with herpesvirus meningitis/encephalitis based on cerebrospinal fluid mNGS positivity

**DOI:** 10.3389/fneur.2026.1808867

**Published:** 2026-05-14

**Authors:** Hongyan Yang, Libin Zhao

**Affiliations:** 1 Department of Neurology, Shenzhen People's Hospital (The First Affiliated Hospital, Southern University of Science and Technology; The Second Clinical Medical College, Jinan University), Shenzhen, China; 2 Department of Anethesiology, Shenzhen Baoan Women's and Children's Hospital, Shenzhen, China

**Keywords:** cerebrospinal fluid, clinical features, encephalitis/meningitis, herpesvirus, metagenomic next-generation sequencing, outcomes

## Abstract

**Background:**

Herpes viruses are a major cause of meningitis/encephalitis in adults. However, their individual clinical phenotypes and outcomes remain incompletely delineated. Metagenomic next-generation sequencing (mNGS) of cerebrospinal fluid (CSF) offers a powerful tool for precise pathogen identification, facilitating the comparison of distinct herpes virus infections.

**Methods:**

This retrospective cohort study analyzed 66 patients with CSF-mNGS confirmed herpes virus meningitis/encephalitis at a single center between October 2019 and August 2025. The cohort was stratified into five etiological groups: herpes simplex virus type 1 (HSV-1, *n* = 10), herpes simplex virus type 2 (HSV-2, *n* = 5), varicella-zoster virus (VZV, *n* = 27), Epstein–Barr virus (EBV, *n* = 15), and human herpesvirus 7 (HHV-7, *n* = 9). Demographic, clinical, laboratory, and neuroimaging data were collected. Outcomes were assessed using the Glasgow Outcome Scale (GOS) at 3 months post-discharge.

**Results:**

Distinct clinical phenotypes were observed. HSV-1 encephalitis typically presented with psychiatric symptoms, seizures, and temporal lobe involvement on MRI. HSV-2 infection manifested primarily as a febrile headache syndrome with minimal brain parenchymal involvement. VZV infection was associated with the most intense CSF inflammatory response (highest WBC and protein), a higher incidence of hypoglycorrhachia (25.9%) and hypochloridia (40.7%), and unique complications like cranial neuritis and vasculopathy. EBV infections occurred in older patients and showed features overlapping with HSV-1. HHV-7 infected a significantly younger population and was strikingly associated with elevated intracranial pressure (ICP ≥ 330 mmH_2_O in 33.3%). Multivariate analysis identified a longer interval from symptom onset to hospitalization (OR: 1.118, *p* = 0.025) and an abnormal EEG (OR: 0.066, *p* < 0.001) as independent predictors of an unfavorable outcome (GOS < 5). Antiviral or steroid therapy was not significantly associated with prognosis in this cohort.

**Conclusion:**

CSF-mNGS reveals distinct and clinically significant phenotypic differences among various herpesvirus meningitis/encephalitis. VZV is characterized by a vigorous CSF inflammatory response and vascular complications, while HHV-7 predominantly affects younger adults and is significantly associated with intracranial hypertension. These findings underscore the value of mNGS in enabling pathogen-directed diagnosis and management, moving beyond syndromic approaches.

## 1 Introduction

Viruses are a major cause of meningitis/encephalitis in adults in China and Western countries (1, 2). However, the causative pathogen remains unidentified in 50–60% of clinically diagnosed viral meningitis cases (3, 4). Although viral meningitis/encephalitis is often considered a benign disease, a considerable proportion of patients do not fully recover (5–7). Unidentified pathogens can lead to excessive diagnostic testing, potentially prolonged treatment and hospitalization, and the prognosis for these patients remains unclear.

The diagnostic paradigm for infectious diseases has traditionally relied on targeted PCR, which offers high sensitivity and specificity for confirming suspected pathogens (8). A critical limitation of this method, however, is its inherent dependence on clinical presumption of the causative agent (9). Metagenomic next-generation sequencing (mNGS) represents a shift in this paradigm. As a hypothesis-free approach, mNGS can comprehensively identify a wide array of pathogens (viruses, bacteria, fungi, and parasites) from a single CSF sample without the need for prior suspicion (10, 11). This capability is crucial for detecting unconventional, novel, or co-infecting pathogens that fall outside standard PCR panels, ultimately minimizing diagnostic delays and facilitating earlier initiation of pathogen-directed therapy.

In China, the incidence of meningitis/encephalitis caused by herpes viruses is relatively high (12, 13). Clinical features and prognosis may differ among different types of herpes viruses (7, 14). Patients may experience long-term health issues after infection, such as seizures and cognitive decline (6, 15), and it can even lead to fatal encephalitis syndromes. Therefore, early and accurate diagnosis and effective treatment are crucial for improving patients’ quality of daily life and survival rates. In-depth research in this field helps promote the widespread clinical application of mNGS and ultimately improve treatment outcomes and overall prognosis for patients with central nervous system infections.

Based on the current research landscape, this study collected case data from inpatients at Shenzhen People’s Hospital over the past nearly 6 years (October 2019–August 2025) who were diagnosed with herpes virus meningitis/encephalitis via CSF mNGS, summarized clinical characteristics, and analyzed disease prognosis. It also explored the practical impact of mNGS testing on disease management.

## 2 Materials and methods

### 2.1 Study subjects

This retrospective study collected clinical data from patients diagnosed with herpes virus meningitis/encephalitis via CSF mNGS at our hospital between October 2019 and August 2025. The study was approved by our hospital’s ethics committee and conducted in accordance with the 1964 Declaration of Helsinki.

Inclusion criteria: ① Age 14 years and older; ② Complete and reliable medical history, with relevant laboratory tests and examinations available. ③ CSF underwent NGS testing. ④ Herpes virus was detected in CSF by NGS. ⑤ Judged by clinicians, the herpes virus was the causative pathogen. Exclusion criteria: ① Final diagnosis confirmed as another condition (e.g., bacterial, fungal, tuberculosis infection, paraneoplastic syndrome, vascular disease). ② Key clinical data or follow-up data severely missing, preventing outcome evaluation. ③ Pregnancy. ④ Mixed infections (patients with cerebrospinal fluid (CSF) mNGS results indicating multiple pathogens and an inability to ascertain the primary causative agent based on clinical characteristics).

### 2.2 Clinical data

We meticulously collected comprehensive patient data, including demographic characteristics such as age and gender, as well as comorbidity (especially history of diabetes, rheumatic immune system diseases, hematological diseases, etc.), and systematically recorded patients’ clinical manifestations and physical examination findings to fully understand their condition.

We also carefully recorded admission time, time interval from symptom onset to admission, and time interval from symptom onset to lumbar puncture for accurate temporal analysis. Laboratory tests included CSF analysis, CSF mNGS results, cranial imaging results, and electroencephalography (EEG) to obtain necessary baseline data. Treatment interventions, including antiviral medications, corticosteroids, and immunoglobulin administration and course, were recorded. Clinical outcomes were strictly assessed, with particular emphasis on length of hospital stay and complications. During follow-up assessments, prognosis was evaluated using the Glasgow Outcome Scale (GOS) score at 1 month post-discharge and 3 months post-discharge to provide a comprehensive view of the patient’s recovery process.

The Glasgow Outcome Scale (GOS) classifies patient outcomes into five categories (details Table 1): death, vegetative state, severe disability requiring daily assistance, moderate disability with independent living capacity, and good recovery with no or only mild sequelae. Outcome assessments were conducted at discharge, as well as at the 1-month and 3-month follow-up visits after treatment. A GOS score of 4 or lower was defined as a poor prognosis, while a score of 5 was considered indicative of a favorable prognosis.

**Table 1:** Details the specific scoring criteria of the Glasgow outcome scale (GOS).

GOS score	Outcomes category	Description
1	Death	–
2	Vegetative state	Unconscious with basic physiological functions preserved
3	Severe disability	Dependent on others for daily living
4	Moderate disability	Independent living with functional limitations
5	Good recovery	No disability or only minor disability

### 2.3 Cerebrospinal fluid mNGS testing

For all patients clinically suspected of central nervous system infection, cerebrospinal fluid (CSF) samples were collected via lumbar puncture under aseptic conditions prior to the initiation of empirical antiviral/antibacterial therapy. The initial CSF sample was preferentially sent to the microbiology laboratory for routine, biochemical, and culture analyses. Subsequently, the remaining 1–2 mL of CSF was aliquoted and stored at −80 °C, with transport to the sequencing laboratory required within 2 h at 4 °C, where it was held until mNGS analysis was performed.

#### 2.3.1 Metagenomic next-generation sequencing of CSF samples

All CSF samples, with a volume of ≥800 μL, were stored at 4 °C and delivered to the laboratory within 2 h post-collection. For viscous samples with a cell count >1 × 10^6^ cells/mL, a liquefaction step was performed using an equal volume of dilution buffer for 5 min. If the sample exhibited an excessively high cell count (>2 × 10^7^ cells/mL) or significant viscosity, a host DNA depletion step was implemented: following a 10-min incubation with a release reagent at 37 °C, the sample was washed with phosphate-buffered saline (PBS) and finally resuspended in 800 μL of PBS.

Subsequently, 600 μL of the processed sample was transferred to a Lysing Matrix E tube for mechanical disruption using a homogenizer at a speed of 6 m/s for two cycles of 45 s each. After centrifugation, the supernatant was collected for total DNA extraction. DNA extraction was performed automatically using the GeneRotex 48 system with the GensKey 2005 magnetic bead-based method, resulting in a final elution volume of 65 μL. The extracted DNA was quantified using a Qubit fluorometer (Thermo Fisher Scientific). The DNA concentration of samples subjected to host depletion typically did not exceed 2 ng/μL.

Library construction was performed using 3–30 ng of DNA. The procedure included: enzymatic fragmentation and end repair (37 °C for 20 min, 72 °C for 20 min), adapter ligation (20 °C for 15 min), and purification using 0.6 × magnetic beads. This was followed by index PCR amplification (12 cycles for standard samples; 16 cycles for host-depleted samples). The amplification products were recovered using 0.9 × magnetic beads, and the final library was eluted in 42.5 μL of EB buffer. A qualified final library required a concentration of ≥1 ng/μL with a primary fragment peak around 300 bp. Sequencing was conducted on an Illumina NovaSeq 6000 platform employing a PE150 strategy (paired-end sequencing, 150 bp reads), ensuring a minimum of 20 million raw reads per sample.

#### 2.3.2 Bioinformatic analysis and pathogen identification

The raw sequencing data were demultiplexed using the bcl2fastq software. Subsequently, Trimmomatic was employed for quality control filtering to remove low-quality reads. High-quality reads were aligned to the human reference genome (version hg38) using the Burrows-Wheeler Aligner (BWA), effectively removing ≥95% of human-derived sequences.

The remaining microbial sequences were taxonomically classified using Kraken2 (with a standard database integrated with specialized databases for fungi, viruses, and mycobacteria), and Bracken was utilized for abundance correction at the species level by estimating read counts. This study implemented stringent positive reporting thresholds:

For DNA viruses: the requirement was a species-specific read count ≥3 and a reference genome coverage ≥20%.

Furthermore, the confirmation of a positive result required meeting the following quality control criteria: the detection rate of the pathogen was <5% in a background microbial database constructed from 50 bronchoalveolar lavage fluid samples of healthy individuals; the read count for the Depletion Negative Control (DNC) in the test sample was <10; no definitive pathogen was detected in the No Template Control (NTC); and the deviation in species composition for the ZymoBIOMICS Microbial Community Standard, used as a positive control, was less than one order of magnitude compared to its known standard.

## 3 Statistical analysis

All statistical analyses were performed using SPSS software (SPSS 20.0). Baseline demographic data were described using frequency distributions. Continuous non-parametric data were analyzed using medians and interquartile ranges (IQRs). Continuous variables conforming to a normal distribution were expressed as mean ± standard deviation.

## 4 Results

**Figure 1:**
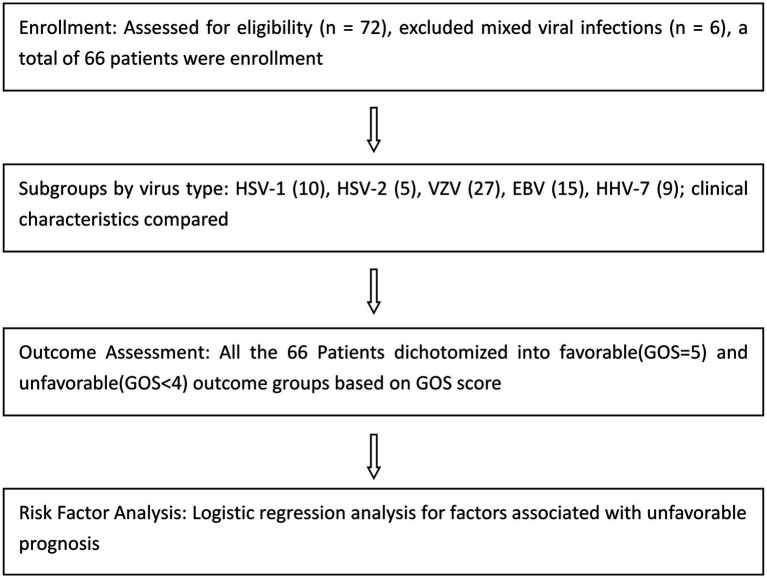
CONSORT flow diagram of patient selection and grouping.

A total of 72 patients with suspected herpesvirus meningitis/encephalitis were assessed for eligibility. After excluding six cases with mixed viral infections, 66 patients with confirmed herpesvirus infection by cerebrospinal fluid metagenomic next-generation sequencing (mNGS) were ultimately included. Based on the detected pathogens, patients were stratified into five subgroups: HSV-1 (*n* = 10), HSV-2 (*n* = 5), VZV (*n* = 27), EBV (*n* = 15), and HHV-7 (*n* = 9). Clinical characteristics were compared among these subgroups. Subsequently, all 66 patients were dichotomized into favorable and unfavorable outcome groups according to their Glasgow Outcome Scale (GOS) scores. Finally, logistic regression analysis was performed to identify factors associated with unfavorable prognosis. The detailed flow diagram is shown in Figure 1. Table 2 is a summary of the characteristics of each group in terms of epidemiology, clinical presentation, and laboratory findings.

**Table 2:** Comparison of clinical features of five different herpesvirus meningitis/encephalitis.

Items	HSV-1 (*n* = 10)	HSV-2 (*n* = 5)	VZV (*n* = 27)	EBV (*n* = 15)	HHV-7(*n* = 9)
Baseline characteristics					
Age (year) (Median, IQR)	42.6 (36.5, 18)	38 (31, 21)	37.9 (34, 14)	44.7 (49, 30)	26.6 (27, 9)
Female (*n* = *x*) (%)	5 (50.0)	1 (20.0)	14 (51.9)	7 (46.7)	6 (66.7)
Immunosuppressive therapy (%)	/	/	2 (7.4)	2 (13.3)	1 (11.1)
Clinical features					
Fever (%)	7 (70.0)	5 (100)	23 (85.2)	12 (80.0)	9 (100)
Headache (%)	6 (60.0)	4 (80.0)	20 (74.1)	6 (40.0)	6 (66.7)
Characteristic rash (%)	1 (10.0)	1 (25.0)	7 (25.9)	2 (13.3)	/
Meningeal irritation sign (%)	3 (30.0)	2 (40.0)	10 (37.0)	4 (26.7)	3 (33.3)
Impaired consciousness (%)	1 (10.0)	/			1 (11.1)
Psychiatric symptoms (%)	2 (20.0)	/	/		2 (22.2)
Seizures (%)	3 (30.0)	/	/	4 (26.7)	2 (22.2)
Focal neurological deficit (%)	3 (30.0)	/		3 (20)	
Cranial nerves and peripheral nerves involvement (%)	/		3 (11.1)	/	
Abnormalities in neuroimaging and electroencephalography (EEG) findings					
Abnormal MR (%)	3 (30.0)	1 (20.0)	3 (11.1)	2 (13.3)	3 (33.3)
Temporal lobe involvement (%)	3 (30.0)	1 (20.0)	/	2 (13.3)	2 (22.2)
Limbic system involvement (%)	3 (30.0)	/		2 (13.3)	/
Multifocal intracranial lesions (%)	/	/	2 (7.4)		1 (11.1)
Vasculopathy (%)	/	/	2 (7.4)	/	/
Abnormal EEG (%)	3 (30.0)	/	4 (14.8)	2 (13.3)	2 (22.2)
Focal slow waves/epileptic waves (%)	3 (30.0)		/	2 (13.3)	1 (11.1)
Diffuse slow waves (%)	2 (20)		1 (3.7)	2 (13.3)	/
Changes in CSF					
WBC Mean (Median, IQR) (×10^6^/L)	130 (32, 234)	336.8 (244, 306)	309.9 (290, 389)	213 (110, 284)	102 (137.9, 266)
Predominantly monocytic (%)	9 (90.0)	5 (100)	27 (100)	15 (100)	9 (100)
Protein Mean (Median, IQR) (g/L)	0.61 (0.55, 0.61)	0.75 (0.68, 0.44)	1.06 (0.91, 0.87)	0.72 (0.57, 0.76)	0.75 (0.53, 0.93)
Pressure ≥ 300mmH_2_O (*n* = *x*) (%)	1 (10.0)	1 (20.0)	6 (22.2)	2 (13.3)	3 (33.3)
Pressure ≥ 330mmH_2_O (*n* = *x*) (%)	1 (10.0)	0 (0.0)	4 (14.8)	2 (13.3)	3 (33.3)
Glucose < 2.5 mmol/l	1 (0.0)	1 (20.0)	7 (25.9)	3 (20.0)	0 (0.0)
Chloride < 120 mmol/l	2 (20)	2 (40.0)	11 (40.7)	7 (46.7)	2 (22.2)

IQR: interquartile range, CSF: cerebrospinal fluid.

### 4.1 Demographics and underlying conditions

**Figure 2:**
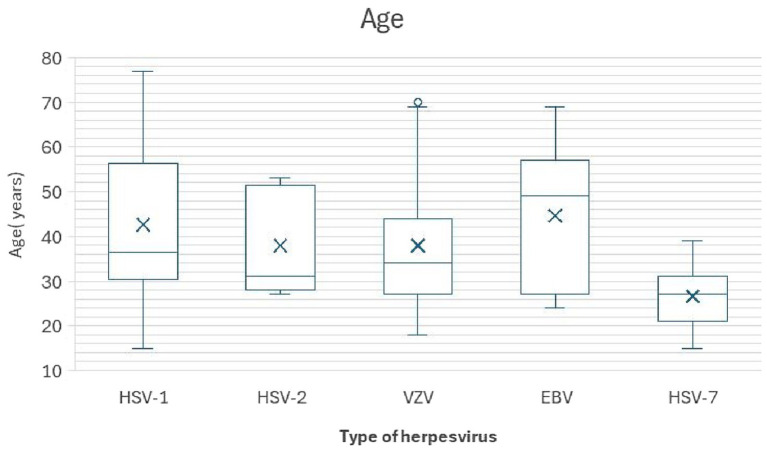
Age distribution of patients with mNGS-positive herpesvirus CNS infections. The mean age, median, and interquartile range (IQR) for each viral group are depicted. Data are presented as mean (median, IQR). Patients in the HHV-7 group exhibited a notably lower mean age of onset (26.6 years) compared to other herpesvirus types.

Box plots Figure 2 show the distribution of age for patients in each mNGS-confirmed herpesvirus group.

### 4.2 Age characteristics

Figure 2 shows the age distribution of five groups.

The HHV-7 group had the lowest mean age (26.6 years, Median 27, IQR 9), while the mean ages of the other groups ranged from 37.9 to 44.7 years. All groups showed a male predominance, particularly the HHV-7 (66.7%) and HSV-1 (50.0%) groups. Immunosuppression was relatively uncommon, with the highest proportion in the EBV group (13.3%), followed by HHV-7 (11.1%) and VZV (7.4%) groups.

Statistical evaluation using one-way ANOVA and post-hoc tests indicated that the mean age of onset in the HHV-7 group was significantly lower than that in the HSV-1 group (younger by 16.044 years, *p* = 0.026) and the EBV group (younger by 18.111 years, *p* = 0.007). This distribution pattern suggests a unique age predisposition for HHV-7-associated CNS infections.

### 4.3 Clinical features

#### 4.3.1 Clinical symptoms

Fever was a universal manifestation, present in all HSV-2 and HHV-7 infected patients, and highly prevalent in VZV (85.2%) and EBV (80.0%) cases. Headache was common in most groups but relatively less frequent in EBV patients (40.0%). Rash was characteristically associated with VZV infection (25.9%). The proportion of patients with meningeal signs ranged from 26.7 to 40.0% across groups.

Neurological manifestations varied significantly. HSV-1 infection was associated with higher rates of focal neurological deficits (30.0%), seizures (30.0%), and psychiatric symptoms (20.0%). HSV-2 meningitis presented predominantly as a febrile headache syndrome with a low incidence of severe neurological complications. VZV infection was closely associated with cranial and peripheral nerve involvement (11.1%). Seizures occurred in both EBV (26.7%) and HHV-7 (22.2%) infections, with the latter also showing impaired consciousness (11.1%) and psychiatric symptoms (22.2%).

#### 4.3.2 CSF characteristics

**Figure 3:**
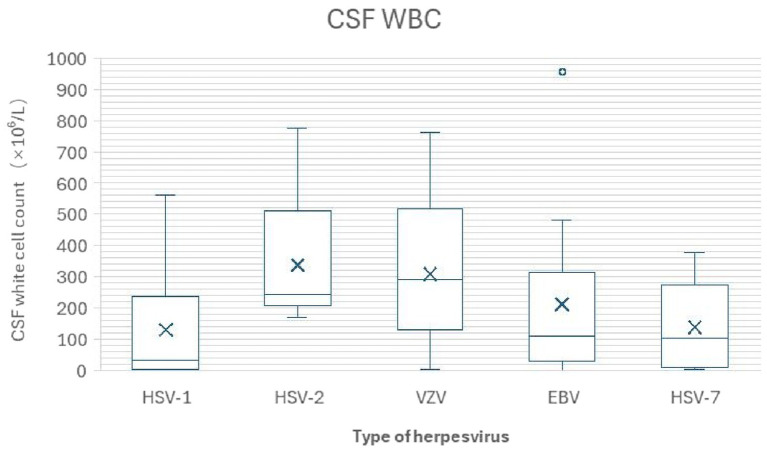
Cerebrospinal fluid white blood cell (WBC) counts across herpesvirus subtypes. The WBC counts varied significantly among groups. The VZV group exhibited the highest median count (290 × 10^6^/L) with the widest distribution, whereas the HSV-1, EBV, and HSV-7 groups showed lower and more concentrated values. Post-hoc comparisons confirmed that the VZV group had significantly higher mean WBC counts compared to the HSV-1 and HSV-7 groups (both *p* < 0.05). In Figures 2, 3: The box represents the interquartile range (IQR, 25th to 75th percentile), the horizontal line inside the box represents the median, the cross mark (×) represents the mean. The whiskers extend to the minimum and maximum values within 1.5 times the IQR from the quartiles, and individual points outside the whiskers represent outliers.

Cerebrospinal fluid white blood cell (WBC) counts exhibited significant differences among patients infected with different herpesvirus subtypes. The mean values (median, interquartile range) for the HSV-1, HSV-2, VZV, EBV, and HSV-7 groups were 130 (32, 234), 336.8 (244, 306), 309.9 (290, 389), 213 (110, 284), and 102 (137.9, 266) × 10^6^/L, respectively, which shows in Figure 3. Overall, the HSV-3 group showed the highest median WBC count (290 × 10^6^/L) and the widest data distribution range (interquartile range spanning 389). In contrast, the HSV-1, EBV, and HSV-7 groups displayed lower median WBC counts with more concentrated data distributions. Post-hoc analysis following one-way ANOVA revealed significant differences in the degree of cerebrospinal fluid pleocytosis among the subtypes (*p* < 0.05). Specifically, the mean cerebrospinal fluid WBC count in the VZV (varicella-zoster virus) group was significantly higher than that in the HSV-1 group (*p* = 0.025) and the HSV-7 group (*p* = 0.038), suggesting that VZV infection may elicit a more intense intrathecal inflammatory response.

CSF characteristics showed distinct patterns. The HSV-2 and VZV groups had the most pronounced inflammatory response, with the highest mean CSF white blood cell counts (HSV-2: 336.8 × 10^6^/L; VZV: 309.9 × 10^6^/L) and significantly elevated protein levels (VZV: 1.056 g/L; HSV-2: 0.75 g/L). The lymphocyte proportion was significantly elevated in all groups. CSF hypoglycorrhachia (CSF glucose < 2.5 mmol/L with CSF/blood glucose ratio < 0.5) was most common in the VZV (25.9%) and HSV-2 (20.0%) groups. Hypochloridia (chloride < 120 mmol/L with CSF/blood chloride ratio < 1.3) was frequent across groups, particularly in the EBV (46.7%) and VZV (40.7%) groups. Markedly elevated intracranial pressure (≥300 mmH₂O) was also a notable feature in HHV-7 (33.3%) and VZV (22.2%) infections.

Post-hoc analysis of one-way ANOVA found significant variation in the degree of CSF pleocytosis. Specifically, the VZV group’s mean CSF WBC count was significantly higher than that of the HSV-1 group (*p* = 0.025) and the HHV-7 group (*p* = 0.038), indicating a more intense intrathecal inflammatory response in VZV-related cases.

For other clinical parameters-intracranial pressure, CSF protein, CSF glucose, and CSF chloride-no statistically significant differences were found among the groups. This may indicate that while different herpes virus infections differ in clinical presentation and patient characteristics, these other parameters are not major sources of variation among these infection types.

#### 4.3.3 Neuroimaging and electrophysiological findings

Brain MRI abnormalities (temporal lobe lesions, limbic system lesions, multifocal lesions, vascular lesions) were most frequently observed in the HHV-7 (33.3%) and HSV-1 (30.0%) groups. Involvement of the temporal lobe and limbic system was characteristic of HSV-1 encephalitis (both 30.0%). EBV infection also demonstrated temporal lobe (13.3%) and limbic system (13.3%) involvement. In contrast, VZV-associated neuroimaging findings were heterogeneous, including cases suggestive of vasculitis or cerebral infarction.

**Figure 4:**
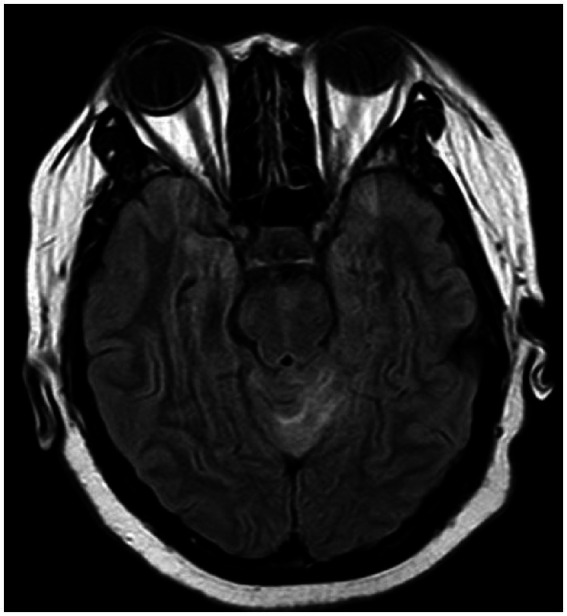
Patchy hyperintensities on T2-weighted FLAIR imaging in the left cerebellar hemisphere and adjacent vermis, suggestive of viral encephalitis.

**Figure 5:**
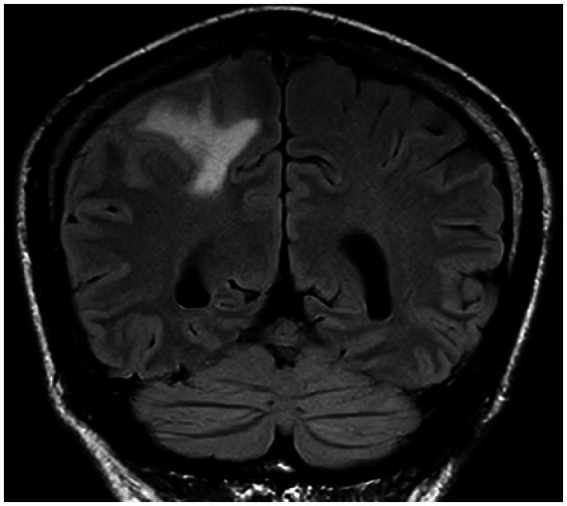
Signal alterations in the right parietal lobe (T2/FLAIR-hyperintense) suggestive of an infectious process.

**Figure 6:**
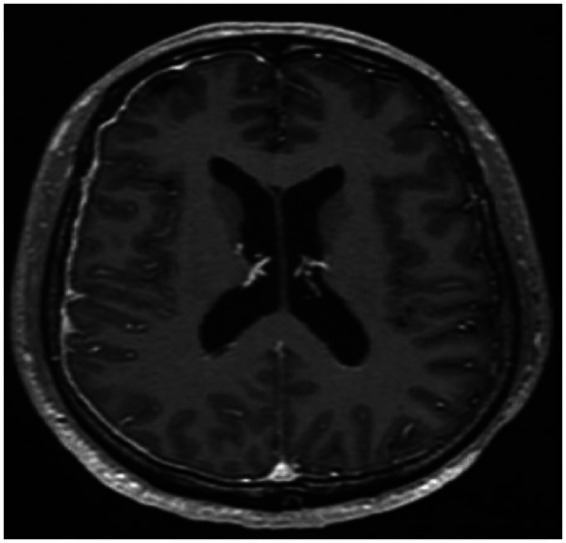
Leptomeningeal thickening over the right cerebral hemisphere, consistent with an infectious etiology.

**Figure 7:**
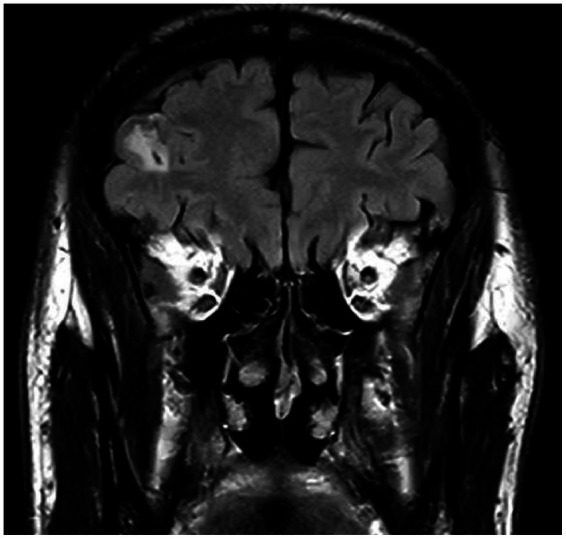
Right frontal lobe lesions demonstrating mild hypointensity on T1-weighted imaging and hyperintensity on T2-weighted/FLAIR sequences, consistent with an infectious process.

**Figure 8:**
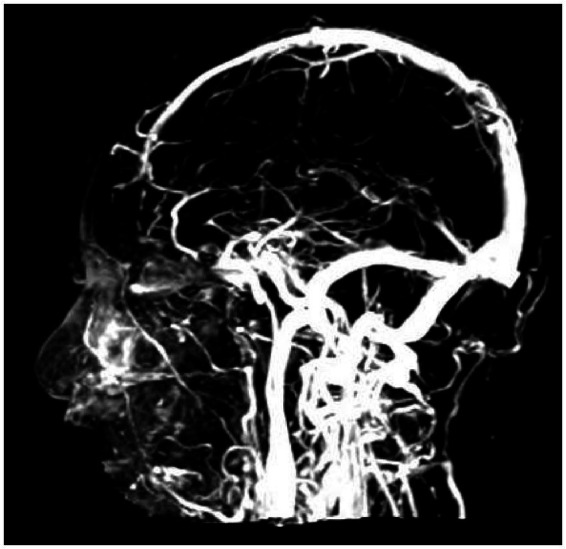
Magnetic resonance venography (MRV) shows nodular hyperintense filling defects within the superior sagittal sinus and straight sinus (arrows), suggestive of venous sinus thrombosis.

In patients with herpes zoster virus encephalitis/meningoencephalitis, neuroimaging revealed diverse abnormalities including a right frontoparietal lesion; meningeal enhancement; cerebellar hemorrhage; and notably, cerebral venous sinus thrombosis. Representative imaging findings are detailed in Figures 4–8.

Electroencephalogram (EEG) abnormalities, including focal slowing, diffuse slowing, and epileptiform discharges, were identified in all groups with available data, with the highest incidence observed in the HHV-7 group (22.2%). Focal slowing or epileptiform discharges, particularly within the temporal regions, were characteristic features associated with HSV-1 infection.

### 4.4 Prognosis

Using the Glasgow Outcome Scale (GOS) scores at 1 month and 3 months post-discharge as the outcome measures (where a GOS score <5 was defined as an unfavorable outcome, and a score of 5 as a favorable outcome). Table 3 details the specific etiologies and corresponding GOS scores for the 13 patients who exhibited an unfavorable outcome at both 1 month and 3 months after discharge. Clinical characteristics were compared between the favorable outcome group (*n* = 53) and the unfavorable outcome group (*n* = 13), as presented in Table 4. Continuous variables are presented as median (interquartile range, IQR). Categorical variables are presented as number (%). Compared to the favorable outcome group, patients in the unfavorable outcome group had a significantly longer onset-to-admission time (median7, IQR18) days vs. (median5, IQR4) days, a higher proportion of immunoglobulin therapy (30.8% vs. 5.7%), a higher rate of corticosteroid use (92.3% vs. 58.5%), and a significantly higher incidence of abnormal electroencephalogram (EEG) findings (61.5% vs. 5.4%).

**Table 3:** Etiology and GOS scores of patients with persistent unfavorable outcomes.

Type	GOS at 1 month post-discharge	GOS at 3 months post-discharge
HSV-1	3	4
HSV-1	4	4
HSV-1	4	4
HSV-1	3	3
VZA	3	4
VZA	3	4
VZA	4	4
EB	4	4
EB	3	3
EB	3	3
EB	3	4
HHV-7	4	4
HHV-7	3	4

**Table 4:** Comparison of clinical characteristics between patients with favorable and unfavorable outcomes in herpes viral encephalitis/meningitis.

Clinical characteristics	Favorable outcome group (53 patients)	Unfavorable outcome group (13 patients)
Onset-Admission time (days) (Median, IQR)	(5, 4)	(7, 18)
Antiviral therapy (%)	52 (98.1%)	13 (100%)
Initiation of antiviral therapy >48 h after admission (%)	12 (22.6%)	2 (15.4%)
Immunoglobulin therapy (%)	3 (5.7%)	4 (30.8%)
Presence of comorbid immunosuppression (%)	7 (13.2%)	2 (15.4%)
Corticosteroid therapy (%)	31 (58.5%)	12 (92.3%)
Abnormal electroencephalogram (EEG) finding (%)	5 (5.4%)	8 (61.5%)

Binary logistic regression analysis was performed on the 66 patients to identify independent predictors of an unfavorable prognosis. The results confirmed that a longer interval from symptom onset to hospital admission (OR: 1.118, 95% CI: 1.014–1.233, *p* = 0.025) and an abnormal electroencephalogram (EEG) finding (OR: 0.066, 95% CI: 0.014–0.315, *p* < 0.001) were significant independent predictors of an unfavorable outcome. In contrast, antiviral therapy, time to initiation of antiviral therapy, the presence of comorbid immunosuppression (*p* = 0.732), and corticosteroid therapy (*p* = 0.517) showed no significant association with prognosis in this study cohort.

## 5 Discussion

This study describes the distinct clinical phenotypes of meningitis/encephalitis caused by different species of herpes viruses, all identified through CSF mNGS. Our findings highlight significant differences in demographic susceptibility, clinical presentation, neuroimaging correlates, and CSF features, which have profound implications for diagnosis, treatment, and prognosis.

### 5.1 A summary and comparative findings of encephalitis/meningitis caused by five herpesviruses

HSV-1 encephalitis most closely fits the classic definition of “viral encephalitis.” The acute triad of psychiatric symptoms, seizures, and temporal lobe abnormalities on imaging is highly specific for diagnosing HSV-1 encephalitis, representing a typical “limbic encephalitis.” The high incidence of psychiatric symptoms (20%), focal deficits (30%), and specific MRI findings (temporal lobe involvement 30%, limbic system involvement 30%) in the HSV-1 group in this study further solidify this prototype.

Although the MRI abnormality rate in this group (30%) may seem low, this could be related to the timing of the examination being too early. Conversely, a normal MRI cannot rule out HSV-1 encephalitis, but once MRI abnormalities are present and located in the temporal lobe, the diagnostic specificity is extremely high. The “focal temporal slow waves/epileptiform waves” on EEG highly correlate with imaging findings and serve as important auxiliary evidence.

HSV-2 meningitis presents a distinctly different syndrome. The absence of parenchymal manifestations such as seizures or focal deficits, combined with a 100% fever rate and meningeal inflammatory signs (80% headache, 40% meningeal irritation), underscores its primary meningeal tropism. This aligns with the clinical characterization of HSV-2 as a cause of benign lymphocytic meningitis described in the literature (16, 17). In our study, a higher incidence of hypoglycorrhachia was observed in HSV-2 meningitis patients. Previous literature has reported acute myelitis with hypoglycorrhachia and elevated protein in CSF associated with HSV-2 infection (18), though the underlying mechanism remains unclear. All five patients with HSV-2 encephalitis/meningitis in this study were cases of primary infection. Due to the lack of long-term follow-up in this study, a tendency for recurrence was not observed.

VZV infection demonstrated the most heterogeneous presentation of CNS involvement. Apart from the characteristic rash (25.9%), it was the only virus significantly associated with cranial and peripheral nerve damage (11.1%), reflecting its neurotropism. Our study observed that compared to other herpesvirus infections, patients with VZV encephalitis/meningitis exhibited more pronounced CSF inflammatory responses, more frequent vasculopathy, and were accompanied by a higher incidence of hypoglycorrhachia and hypochlororrhachia. This highlights VZV’s propensity to cause vasculopathy rather than pure encephalitis (14, 19, 20). This unique CSF profile likely stems from VZV’s specific pathogenic mechanisms.

Multiple studies suggest that during CNS infection, Varicella-Zoster Virus (VZV) not only directly invades neurons and glial cells but can also infect vascular wall cells, triggering vasculitis and vascular remodeling, thereby exacerbating the inflammatory response.

Research by Nagel et al. (21) indicated that following VZV infection of arteries, early infiltration of numerous neutrophils, T cells, and macrophages occurs in the adventitia, accompanied by viral antigen deposition. Vascular pathology and blood–brain barrier (BBB) disruption lead to a significant influx of inflammatory cells (primarily lymphocytes) into the CSF, directly contributing to elevated CSF white cell count and protein concentration. Pathological changes such as intimal thickening, elastic fiber fragmentation, and smooth muscle cell loss are closely associated with vascular stenosis, ischemic, or hemorrhagic events (21).

Further research (22) found that VZV-infected human brain vascular adventitial fibroblasts (HBVAFs) showed activation of transcriptional pathways related to vascular remodeling and amyloidosis at the transcriptome level, including upregulation of MMP-3 and MMP-10 expression, promoting cell migration and extracellular matrix degradation. More notably, VZV infection can induce the expression of Aβ42 and amylin, leading to the formation of intracellular amyloid deposits (22). This may be a key mechanism underlying persistent inflammation and tissue damage in VZV vasculopathy.

Mescher et al. (2021), in autopsies of two patients with cerebral amyloid angiopathy (CAA), found VZV antigens and DNA co-localized with Aβ in some affected arteries, suggesting VZV infection might promote or accelerate amyloid deposition in cerebral blood vessels, thereby worsening vascular inflammation and dysfunction (23).

In summary, VZV infection may induce vasculopathy and an exacerbated CSF inflammatory response through multiple mechanisms: direct invasion of the vascular wall, induction of inflammatory cell infiltration, promotion of vascular remodeling, and amyloid deposition. This leads to the observed phenomena of increased cells and elevated protein levels in the CSF of our VZV patients.

This is fully reflected in VZV’s characteristic CSF pattern of “hypoglycorrhachia, hypochlororrhachia, and elevated protein.” This finding not only aids clinicians in prioritizing VZV etiology based on CSF analysis but also provides a theoretical basis for combined antiviral and anti-inflammatory therapy.

EBV-related CNS infections present complex features. Although it shares some characteristics with HSV-1 (e.g., limbic system involvement 13.3%, seizures 26.7%), its overall clinical presentation is relatively milder in terms of focal deficits. It occurs in a relatively older population (median age 49 years) and has the highest incidence of immunosuppression (13.3%), consistent with recent large-scale studies on EBV-associated encephalitis (24). This indicates it may often manifest as reactivation in susceptible hosts.

Patients in the HHV-7 encephalitis group were the youngest, had a 100% fever rate, and exhibited significant encephalitic features including seizures (22.2%) and psychiatric symptoms (22.2%). There was a high rate of MRI abnormalities (33.3%) and elevated intracranial pressure (ICP) (33.3%). This suggests HHV-7 can cause significant encephalitic disease in young adults. However, all 9 patients had a good 3-month prognosis. Previous literature reports HHV-7 meningitis primarily in children. In our study, the age of onset for HHV-7 infected patients was significantly younger than other groups, consistent with previous reports (25, 26). The high proportion of abnormally elevated ICP (ICP ≥ 330 mmH₂O) observed in our study has not been highlighted in previous reports. The underlying mechanism is likely multifactorial. First, HHV-7 is a lymphotropic virus with high affinity for CD4 + T cells. Its invasion of the CNS may trigger a robust immune-inflammatory response in the subarachnoid space and brain parenchyma. Subsequent release of pro-inflammatory cytokines (such as TNF-*α* and IL-6) can severely disrupt the BBB, leading to significant vasogenic cerebral edema—a major contributor to elevated ICP. Furthermore, some previous reports have found intracranial venous sinus thrombosis in patients (26), suggesting HHV-7 might share a similar pathogenic mechanism with VZV, which requires further investigation.

Although the HHV-7 subgroup sample size was small, the statistical significance of this association underscores its potential clinical relevance. This finding suggests that HHV-7 testing should be considered in patients with encephalitis/meningitis of unknown etiology, especially those presenting with signs of severe intracranial hypertension. Additionally, clinicians should be vigilant about ICP monitoring in confirmed HHV-7 cases, as they may require more aggressive management strategies (such as temporary CSF diversion) to prevent catastrophic neurological deterioration.

Notably, a substantial proportion of patients with viral meningoencephalitis presented with hypoglycorrhachia and hypochloridarchia in the CSF.

Several mechanisms may account for this observation: There are numerous previous reports of hypoglycorrhachia in EBV and VZV encephalitis/meningitis patients (24, 27–29). The pathophysiology of CSF hypoglycorrhachia remains unclear. Possible mechanisms include increased glucose consumption by activated immune cells in the CSF and impaired glucose transport due to BBB dysfunction (30). The angiotropic nature of VZV and EBV makes them prone to inducing granulomatous vasculitis and more severe BBB disruption (30, 31). Concurrently, as a reactivation of a latent virus, VZV infection typically elicits an extremely vigorous cellular immune response (21), leading to a massive influx of highly metabolically active inflammatory cells into the subarachnoid space that competitively consume glucose, thereby causing significant hypoglycorrhachia.

The occurrence of hypochlororrhachia can generally be explained by the Donnan equilibrium effect. Severe BBB disruption caused by viral infection leads to a significant increase in CSF protein levels. To maintain electrical neutrality in the CSF, compensatory shifts of negatively charged chloride ions occur, resulting in a decrease in its concentration (21). Therefore, the observed hypochlororrhachia in this study may serve as an indirect marker of more intense meningeal inflammation and vascular damage.

### 5.2 Discrepancy between pre-admission delay and in-hospital antiviral therapy as prognostic factors

Several factors may explain the seemingly paradoxical finding that delayed hospital admission, rather than the timing of antiviral therapy, emerged as a significant predictor of unfavorable outcomes in this cohort: (1) patients with delayed diagnosis may have already sustained irreversible neurological damage by the time treatment was initiated, a systematic review has demonstrated that a delay in the initiation of antiviral therapy from symptom onset is a critical determinant of poor clinical outcomes in adult patients with herpes simplex virus (HSV) central nervous system (CNS) infection (32); (2) supportive care (e.g., management of intracranial pressure, respiratory support, seizure control) likely plays a crucial role in patient outcomes, and delays in accessing such comprehensive care may contribute to poorer prognosis. These observations underscore the importance of early diagnosis and prompt initiation of both antiviral and supportive therapies.

Virtually, the inherent limitations of a retrospective observational design must be acknowledged. Unlike randomized controlled trials, retrospective studies cannot fully control for unmeasured confounders, including variations in viral load, host immune response, or supportive care quality. Furthermore, the near-universal administration of antiviral therapy in our cohort (with very few untreated patients) limited our ability to compare outcomes between treated and untreated groups, thereby reducing the statistical power to detect a significant treatment effect.

In summary, while antiviral therapy remains the cornerstone of management for herpesvirus CNS infections, our findings highlight the critical importance of the pre-admission phase. Reducing delays in seeking medical care and early diagnosis may be equally important strategies for improving neurological outcomes in this population.

### 5.3 Diagnostic and therapeutic implications of mNGS

Our study strongly demonstrates the utility of CSF mNGS in refining the etiological diagnosis of CNS infections. In cases of suspected viral encephalitis, reliance on syndromic approaches alone is insufficient. For example, patients with seizures and temporal lobe MRI abnormalities are often empirically treated for HSV-1. However, our data indicate that EBV and HHV-7 can present with overlapping features. While acyclovir covers HSV and VZV, it is ineffective against EBV and HHV-7. Therefore, precise identification of HHV-7 or EBV via mNGS can prevent unnecessary long-term acyclovir use and prompt a re-evaluation of treatment strategy.

Consequently, this highlights the indispensable role of pathogen detection in encephalitis diagnosis. For patients with suspected herpesvirus encephalitis but atypical clinical presentations, comprehensive CSF pathogen detection, such as mNGS testing, is essential.

## 6 Study limitations

Our study has several limitations. Due to the scarcity of clinical cases, we did not include patients with cytomegalovirus encephalitis/meningitis, human herpesvirus 6, or human herpesvirus 8 encephalitis/meningitis.

The sample sizes for the five included types of herpesvirus encephalitis/meningitis were small, particularly for the HSV-2 and HHV-7 groups. This limits multivariate analysis and the ability to draw definitive conclusions. Furthermore, as a retrospective observational study, treatment regimens and clinical outcomes were not uniformly assessed, precluding a comparative analysis of prognosis and recovery among the groups. Future large-scale, prospective, multicenter studies are needed to enable analysis of these phenotypes, as well as short-term and long-term outcomes.

## Data Availability

The original contributions presented in the study are included in the article/Supplementary material, further inquiries can be directed to hongyanyang2025@outlook.com.
